# Parental Satisfaction towards Care Given at Neonatal Intensive Care Unit and Associated Factors in Comprehensive and Referral Hospitals of Southern Ethiopia

**DOI:** 10.1155/2023/3338929

**Published:** 2023-08-24

**Authors:** Eden Sileshi, Bedria Mohammed, Derese Eshetu, Aster Dure, Agegnehu Bante, Abera Mersha, Teketel Ermias Geltore

**Affiliations:** ^1^Department of Midwifery, College of Medicine and Health Science, Wachemo University, Durame Campus, Durame, Ethiopia; ^2^Department of Midwifery, Arbaminch College of Health Science, Arba Minch, Ethiopia; ^3^Department of Midwifery, College of Health Science, Madawalabu University, Ethiopia; ^4^School of Nursing, College of Medicine and Health Sciences, Arba Minch University, Arba Minch, Ethiopia

## Abstract

**Background:**

Patient satisfaction is an important aspect of the quality of care in the inpatient setting. In neonatal intensive care units, parents' satisfaction and their experiences are fundamental to assessing clinical practice and improving the quality of care delivered to infants. Hence then, it reduces infant mortality rates globally. In Ethiopia, few studies address the level of parental satisfaction towards care given at neonatal intensive care unit and no single study was done in the study area. Therefore, this study is aimed at assessing parental satisfaction towards care given at neonatal intensive care unit and associated factors in comprehensive and referral hospitals of southern Ethiopia.

**Methods:**

An institutional-based cross-sectional study was conducted among 401 parents who visited neonatal intensive care from March 28 to April 28, 2022. The data were assorted via a structured interviewer-administered questionnaire using ODK collect version and exported to SPSS window version 25 for further cleaning and analysis. Bivariate and multivariate logistic regressions were used to identify factors associated with parental satisfaction with care given at the neonatal intensive care unit. The adjusted odds ratio with 95% CI was used to show the strength of the association, and a *P* value < 0.05 was used to declare the cutoff point to determine the level of significance.

**Results:**

In this study, 63% (95% CI: 58%, 68%) of the parents were satisfied with the care given at the neonatal intensive care unit. Factors associated with parental satisfaction towards care given at neonatal intensive care unit were parents with no formal education (AOR: 0.15; 95% CI: 0.07-0.31), availability of necessary information using direction indicator (AOR: 3.14; 95% CI: 1.85-5.31), and availability of enough chairs in waiting area (AOR: 3.26; 95% CI: 1.81-5.87).

**Conclusion:**

Nearly two-thirds of the parents were satisfied with the care given at the neonatal intensive care unit. The availability of enough chairs in the waiting area and the creation of direction indicators are key issues to improve parental satisfaction towards their neonatal care.

## 1. Background

Patient satisfaction refers to the requirements, expectations, experiences, and assessment of patients with the health care they have received [1, 2]. In the last decade, patient satisfaction has been recognized as a trustworthy and valid tool for assessing the quality of care, which is especially true for hospitalized newborns in critical care units. Parents, as the infant's supporters, are present in the health care system during the neonatal period; therefore, they are the representatives of their infants [3].

The hospitalization of a child would have a tremendous impact on all families' life. These shifts could be the result of family members' concerns about the child's care; they may feel uncomfortable and low-spirited as a result of being in a foreign environment, as well as the possibility of inefficiencies in child care [4]. The neonatal intensive care unit (NICU) is specializing in the care of sick or early newborn infants. So, parents have to come to terms with information that they are not familiar with and have to deal with the emotional impact of the information presented to them [5].

World Health Organization fact sheets 2021 reported that the neonatal period accounted for 47% of all under-five deaths, which is higher compared to the 1990 (40%). With a neonatal death of 27 deaths per 1000 live births, sub-Saharan Africa is the highest in the world followed by southern Asia (with 23 deaths per 1000 live births) [6].

Complications and morbidity are related to a lack of quality care particularly, parental dissatisfaction with care given at the NICU, and lack of professional care and treatment [7]. Parental dissatisfaction results in inefficiency in the care of infants due to a shortage of adequate nursing, medical, and family-centered care [4].

In previous studies conducted in London and Italy, 56% and 87% of the parents were satisfied with the care provided to their newborns, respectively [8, 9]. In a study conducted in Ankara, Turkish mothers are mostly satisfied with the approach of doctors and nurses [4]. A study conducted in Bangladesh showed that 85% of patients were satisfied with the hospital service received. In a study conducted in Tanzania on the quality of neonatal health care, only 9.4% of mothers were satisfied with the amount of time spent by doctors in seeing their babies [10]. A study done in Gondar, Debre Berhan, and Addis Ababa indicates 50%, 77%, and 41.8% of the parents were satisfied by the care given at NICU, respectively [11–13].

Perceiving respect and understanding from health professionals is one of the elements linked to positive parental satisfaction [14]. Maintaining open lines of communication with parents, addressing their questions, and providing the best possible care are all ways to satisfy them in the NICU [4]. Apart from that, being asked for consent and permission before any clinical procedure, being allowed to have visitors, the functionality and cleanliness of the toilets, the quietness of the ward, clear explanations about procedures and medications, and the availability of prescribed medications in the hospital pharmacy were all factors that contributed to good parental satisfaction at neonatal intensive care units [12]. Parents who had their children in a single birth and who had their children outside of the infection isolation area were satisfied [15].

One of the WHO's strategies is to reduce newborn mortality, which is a public health concern. The first step in preventing and lowering newborn morbidity and death is to determine parent satisfaction with neonatal care. Ethiopia has attempted to improve the quality of its health care system by initiating a variety of programs, training health care staff, and educating the general public about health care-seeking behaviors. A few studies were done in Ethiopia that tried to assess factors associated with parental satisfaction towards care given at NICU; each of the studies was done on a single health facility, while this study was done in the hospitals which are found at the regional level, and hence, it is taken as a more representative of the population of the study. At the same time, no single study was conducted in the study area. Therefore, this study is aimed at assessing parental satisfaction and associated factors towards NICU in comprehensive and referral hospitals of southern Ethiopia, in 2022.

## 2. Methods

### 2.1. Setting and Study Design

The current study was employed in the public hospitals of the Southern Nations, Nationalities, and People's Region (SNNPR), which has a total population of 17,887,005 (2021 population census).

The study was conducted at Wolkite comprehensive specialized hospital (WCSH), Dilla University referral hospital (DURH), Wachemo University Nigist Eleni Mohammed memorial referral hospital (WUNEMMRH), and Wolaita Sodo University teaching and referral hospital (WSUTRH) from March 28 to April 28, 2022. Wolkite comprehensive specialized hospital is located in Gurage Zone 158 km southwest of Addis Ababa, the capital city of Ethiopia. The estimated total population of the zone is 28,856. According to hospital records, there were 72 admissions in 2021.

Dilla University referral hospital is found in Dilla City administration which is located 360 km far away from, Addis Ababa, the capital city of Ethiopia. The average monthly admission rate in DURH NICU was 123 in 2021.

WUNEMMRH is found in Hadiya Zone, SNNPR, at 232 kilometers south of Addis Ababa. The hospital gives preventive, curative, and rehabilitative clinical services structured in four case teams outpatient, emergency and critical care, maternal, child, obstetrics, and operation theatre.

Wolaita Sodo University teaching and referral hospital is located in Wolaita Zone, SNNPR, Ethiopia. The hospital is located 332 km south of Addis Ababa and 122 km south of Hawassa. Among the services provided in the hospital are NICU, maternal, neonatal, child health, and kangaroo mother care services.

### 2.2. Study Design

A facility-based cross-sectional study design was employed.

### 2.3. Source Population

All parents whose neonates were admitted to the NICU in comprehensive and referral hospitals of southern Ethiopia were the source population of the study.

### 2.4. Study Population

All parents whose neonates were admitted to the NICU in comprehensive and referral hospitals in southern Ethiopia during the data collection period were the study population.

### 2.5. Eligibility Criteria

All parents with their hospitalized neonates who had a length of stay as inpatients at least 24 hrs in NICU were included in the study.

### 2.6. Exclusion Criteria

Parents of neonates born with major congenital malformations or who died were excluded from the study.

### 2.7. Sample Size Determination and Sampling Technique

The sample size was calculated using single population proportion formula by considering a previous study done at the University of Gondar Comprehensive Specialized Hospital, north Ethiopia, which showed a 50.0% prevalence of parental satisfaction towards care given at the neonatal intensive care unit [11], with 95% confidence interval certainty and 5% confidence limits (degree of precision).

(1) $$\begin{array}{c} n=\frac{\left[z\;{\left(1-\alpha /2\right)}^{2}*p*\left(1-p\right)\right]}{d2}, \end{array}$$

where *n* is the sample size, *p* is the magnitude of parental satisfaction towards NICU service = 50%, *d* is the margin of error of 0.05 with a 95% confidence level, and *Z*_*α*/2_ is the standard normal distribution corresponding to 95% level significance = 1.96.

(2) $$\begin{array}{c} n=\frac{\left(Z\;\alpha /2\right)\;p\left(1-p\right)}{d2}=\frac{\left(1.96\right)2*0.5*\left(0.5\right)}{\left(0.05\right)\left(0.05\right)}=384 \end{array}$$

Then, by adding a 10% nonresponse rate, the total sample size was *n* = 422.

### 2.8. Sampling Procedure

A total of 4 comprehensive and referral hospitals found in the southern region were governed by the south regional health bureau, and those hospitals included were used in the study. The average number of NICU admission in one month was 470 which was taken from the office of the neonatal unit. Then, the calculated sample size was proportionally allocated to each comprehensive and referral hospital in the region. Since the total study population in the selected hospitals is manageable, all of the study populations available during the data collection period were included conveniently in the study.

### 2.9. Data Collection Method

The data were collected by using face-to-face interviewer-administered structured questionnaires, and also, ODK was used to collect data. The questionnaire is partly adapted from previous literature [11, 12, 16–19]. Eight bachelor's degree nurses were recruited as data collectors, and four master's degree nurses were employed as a supervisor.

The data were collected by using face-to-face interviewer-administered structured questionnaires, and also, ODK was used to collect data. The questionnaire is partly adapted from previous literature. The questionnaire contains closed-ended questions. In general, the questionnaire contains seven dimensions: eight sociodemographic-related questions, three parent-related questions, three social support questions, seven neonatal-related questions, eleven pregnancy-related questions, sixteen questions to measure satisfaction towards neonatal care service, and fifteen questions to measure experience toward health service and facilities.

Parental satisfaction was measured by a five-point Likert scale (1, strongly disagree; 2, disagree; 3, not sure; 4, agree; and 5, strongly agree), which was used to measure parental satisfaction status. The items were first categorized into two and then were computed, and they were categorized into satisfied or unsatisfied by taking mean as a cut point. In the previous study conducted in Gondar, the reliability of the data collection tool was checked by Cronbach's alpha which was 0.789.

The social support scale assessed three questions that were taken from the study conducted in Germany. The OSSS-3 consists of only three items that ask for the number of close confidants or intimates, the sense of concern from other people, and the relationship with neighbors with a focus on the accessibility of practical help.

*Data collector*: eight bachelor's degree nurses were recruited as data collectors, and four master's degree nurses were employed as supervisors.

*Procedure:* before the commencement of data collection, data collectors and supervisors were trained for one day on the objective, tools, and process of data collection. All parents with admitted newborns who have stayed 24 hours or above were interviewed by the data collector. The face-to-face interview was conducted with the parents of a neonate in a private room.

### 2.10. Operational Definitions

*Parental satisfaction*: it is defined as attaining the parents' needs or desires. Parental satisfaction was classified into two categories: satisfied and unsatisfied. Overall satisfaction was calculated by summing all items measuring satisfaction and was determined by the cutoff point, which was the mean score. Parents who scored equal to or above the mean score were classified as satisfied with the NICU services, and those who scored below the mean score were reported as unsatisfied [13].

*Social support*: it was measured by the Oslo-3 item social support scale. The sum score ranged from 3 to 14, and the OSSS-3 sum score can be operationalized into three broad categories of social support. Those parents who scored 3–8 were reported as having poor social support, 9–11 were moderate social support, and 12–14 were represented as strong social support [16].

### 2.11. Data Quality Control

The questionnaire was first developed in English and translated into an Amharic version, and it was retranslated back to English to check for its consistency. Both data collectors and supervisors were trained, and pretesting was conducted to ensure the appropriateness of wording and clarity. Internal consistency and reliability of the tool were checked by Cronbach's alpha test. During the data collection, time close supervision and monitoring were carried out by supervisors and the investigator to ensure the quality of the data. The data collectors were responsible to collect data daily, and the supervisor checked the data. The data collectors were from another hospital which was different from their working area and also consistently recorded the result; finally, the result was submitted to the investigator as scheduled. Finally, the collected data was checked by the supervisor and investigator for its completeness.

### 2.12. Data Processing and Analysis

Each data file sent from the data collector's smart mobile phone was downloaded from the server and saved as an Excel file. Finally, the data set was imported to SPSS 25.0 versions for cleaning, coding, and analysis. Descriptive statistics such as frequency, percentage, mean, median, and standard deviation were computed and used for the descriptive part. During analysis, the responses of “very satisfied” and “satisfied” were classified as satisfied and responses of “very dissatisfied,” “dissatisfied,” and “neutral” were classified under unsatisfied. Neutral responses were classified as dissatisfied considering that they may represent a fearful way of expressing dissatisfaction.

Bivariate and multivariable analyses were done in binary logistic regression to identify factors. All assumptions of binary logistic regression were checked. *P* value < 0.25 in the multivariate analysis was considered to take a candidate variable for the final model. The Hosmer-Lemeshow goodness fitness was done to check model fitness. Multicollinearity among independent variables was checked by the variance inflation factor (VIF). The odds ratio with a 95% confidence interval was computed to determine the level of significance. A statistical significance was declared at *P* value < 0.05. The result was presented by using tables and figures.

## 3. Results

### 3.1. Sociodemographic Characteristics

A total of 401 parents participated in the study, resulting in a 95% response rate. Among the participants, three hundred nineteen (79.6%) of the participants were females. The mean age of the participants was 28.68 (S.D ± 5.73) years and the mode of 30 years. Of the participants, 242 (60.3%) were in the age group of 25-35 years. Two hundred five (51.1%) were rural residents, 152 (37.9%) were protestant religious followers, 116 (28.9%) of the respondents did not attend formal education, and thirty-nine (21.4%) of the respondents attended primary education. One hundred thirty-nine (34.7%) of the participants' occupations were housewives, and 371 (92.5%) of the participants were married (Table 1).

### 3.2. Parental-Related Factors

Out of the study participants, 300 (74.8%) were biological mothers of the neonate whereas eighteen (4.7%) of the participants were guardians. Two hundred ninety-eight (74.3%) infants of the parents were breastfed. Three hundred twenty-one (80%) of the parents gave a single birth. One hundred eighty-nine (47.1%) of the parents have strong social support (Table 2).

### 3.3. Neonatal-Related Factors

Two hundred thirty-one (57.6%) of the neonates in the study were male. The minimum and maximum birth weights of the neonates were 1000 grams and 5000 grams, respectively, with an average of 2940.23 (SD, 678.5) grams. The gestational age in weeks at birth was ranging between 27 and 43 weeks with a mean of 37.52 (SD, 2.63) weeks, and 287 (71.6%) were greater than 37 weeks. Regarding the reason for admission, 153 (38.2%) neonates were admitted to the NICU due to neonatal sepsis. Concerning the duration of hospital stay, 290 (72.3%) neonates stayed for 4-14 days (Table 3).

### 3.4. Pregnancy-Related Factors of Parents

One hundred eighty-nine (47.1%) of the participants had a child, 374 (93.3%) of the participants' status of current pregnancy was wanted, and 361 (90.0%) of the participants had ANC follow-up. Regarding the mode of delivery, 274 (68.3%) were delivered by SVD, and 100 (24.9%) of the participants had problems encountered during labor and delivery. Regarding gender of labor attendant, 229 (57.1%) were male (Table 4).

### 3.5. Experience in Health Care Service and Facilities

Among the participating parents, 241 (60%) agreed with the cleanness of the hospital compound and 106 (26.4) parents disagreed with the functionality and cleanness of the hospital. One hundred eight (26.9%) of the parents agreed with the availability of necessary drugs in the pharmacy, and 194 (48.4%) of the parents agreed with the accessibility of information about NICU rules (Table 5).

### 3.6. Parental Satisfaction concerning Neonatal Care

Three hundred ninety-five (98.5%) of the participants were satisfied by respecting confidentiality, and 393 (98.0%) were satisfied by the safety of the NICU environment. Of the total respondents, 385 (96%) were satisfied with counseling about breastfeeding techniques, and 343 (85.5%) were satisfied with explanations about the treatment given at NICU (Table 6).

**Figure 1:**
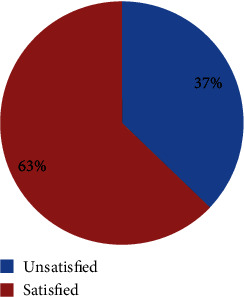
Parental satisfaction status towards neonatal care service at comprehensive and referral hospitals of southern Ethiopia (*n* = 401).

The overall magnitude of parental satisfaction towards neonatal care at the comprehensive and referral hospitals of southern Ethiopia was 63% (95% CI: 58%, 68%) as shown in Figure 1.

### 3.7. Factors Associated with Parental Satisfaction towards Care Given at NICU

In the multivariable analysis, educational status, availability of necessary information using direction indicator, and availability of enough chairs were found to be statistically significant with parental satisfaction towards care given at NICU.

Firstly, parents who had no formal education were 85% less likely to be satisfied with the care than parents who had college and above education (AOR = 0.15; 95% CI: 0.07-0.31). Besides this, the odds of parental satisfaction towards care given at NICU among parents who agreed with the availability of necessary information using direction indicator was 3 (AOR = 3.14; 95% CI: 1.85-5.31). Lastly, the odds of parental satisfaction towards care given at NICU among parents who agreed with the availability of enough chairs in the waiting area was 3 (AOR = 3.26; 95% CI: 1.81-5.87) (Table 7).

## 4. Discussion

This study examined the magnitude of parental satisfaction with NICU service in public hospitals in Ethiopia's SNNPR regional state in 2022, and it showed 63% (95% CI: 58%, 68%). This is in line with the same study done in Black Lion Hospital which was 59.1% [17]. However, it is lower than the other three studies in Ethiopia (80.1% [18], 77% [12], and 74.9% [19]) and in Norway (76% [14]). The reason for the difference might be due to differences in socioeconomic status, ward variation, and variation of the hospital setup-like organization. Moreover, it could be attributed to the year of study which means an increasing number of admission. On the other hand, this finding is higher than the studies conducted in Ethiopia (50% [11], 48% [13], 55% [20], 40.9% [21], and 56.0% [22]) and London (56.0% [8]). The possible explanations for this difference may be due to the suitability to deliver care for clients. Another reason could be due to the study area, as comprehensive and referral hospitals of the southern region. In addition to this, it might be attributed to the quality of the health care system and an increasing number of health professionals in the NICU. The study revealed that no formal education was significantly associated with parental satisfaction. This was consistent with findings done in Kenya [23]. It was contrary to the study done in Norway and Ethiopia [14, 24]. This could be attributed to the fact that parents who had higher educational levels have a better understanding of the care given at NICU and they are more likely to give an accurate assessment of the baby's progress.

The finding of the study showed that agreement with the availability of necessary information using direction indicators was significantly associated with parental satisfaction. This was consistent with the study done in Ethiopia [12]. This could be explained as follows: parents who agreed to the availability of necessary information using direction indicator could get suitable condition to get the place of the NICU service easily, follow their child closely, and take care of their baby until discharge easily. In addition, parents who agreed to direction indicators can complete the treatment of their baby and save time to access the treatment.

This study also demonstrates that agreeing with the availability of enough chairs was significantly associated with parental satisfaction. This was consistent with the study done in Ethiopia [13] and Europe [25]. The possible reason behind this might be that the parents who agreed to the availability of enough chairs in the waiting area had stability and minimum stress about their child's health condition.

### 4.1. Limitations of the Study

This was a cross-sectional study, which cannot determine cause-and-effect relationships. The study was conducted only in a government hospital, and parental satisfaction in a private hospital was excluded. There might have been a possibility of social desirability and recall bias during the interviews.

## 5. Conclusions

According to the result of this study, approximately 63% of the parents were satisfied with the care given at NICU. Educational status, availability of necessary information using direction indicators, and availability of enough chairs were determinant factors for parental satisfaction towards care given at NICU. It is better to provide timely neonatal care services and provide continuous training for health caregivers to improve parental satisfaction towards their neonatal care. Hospital administrators should improve the accessibility of health services to increase quality of care.

## Figures and Tables

**Table 1:** Sociodemographic characteristics of study participants at comprehensive and referral hospitals of southern Ethiopia, 2022 (*n* = 401).

Variables	Frequency	Percentage (%)
Sex		
Male	82	20.4
Female	319	79.6
Educational status		
No formal education	116	28.9
Primary education	86	21.4
Secondary education	92	22.4
Diploma and above	107	26.7
Occupational status		
Government employee	80	20.0
Private employee	82	20.4
Merchant	60	15.0
Farmer	33	8.2
Housewife	139	34.7
Others^¥^	7	1.7
Income(ETB)		
<750	44	11.0
≥750	357	89.0
Marital status		
Single	21	5.2
Married	371	92.5
Divorced	8	2.0
Widowed	1	0.2

^¥^Daily laborer and student.

**Table 2:** Parental-related factors of study participants at comprehensive and referral hospitals of southern Ethiopia, 2022 (*n* = 401).

Variables	Frequency	Percentage (%)
Relationship with the neonate		
Mother	300	74.8
Father	82	20.4
Guardian	19	4.7
Infant breastfeeding		
Yes	298	74.3
No	103	25.7
Type of birth		
Single birth	346	86.3
Multiple births	55	13.7
Social support		
Poor social support	74	18.5
Moderate social support	138	34.4
Strong social support	189	47.1

**Table 3:** Neonatal-related factors at comprehensive and referral hospitals of southern Ethiopia, 2022 (*n* = 401).

Variables	Frequency	Percentage (%)
Neonate's sex		
Male	231	57.6
Female	170	42.4
Birth weight in gram		
<2500	91	22.7
≥2500	310	77.3
Reason for admission of the neonate		
Prematurity with respiratory problem	92	22.7
Neonatal sepsis	153	38.2
Asphyxia	129	32.2
Jaundice	34	8.5
Low blood glucose	24	6.0
Other^€^	19	4.7
Neonate's current health condition		
Good	325	81.0
Bad	76	19.0
Neonate's isolation room admission		
Yes	151	37.7
No	250	62.3
Length of stay in this hospital		
>4	75	18.7
4-14	290	72.3
>14	36	9.0

^€^Cleft palate, hypothermia, severe anemia, and subgaleal hemorrhage.

**Table 4:** Pregnancy-related factors of study participants at comprehensive and referral hospitals of southern Ethiopia, 2022 (*n* = 401).

Variables	Frequency	Percentage (%)
Number of live children (*n* = 189)		
One	44	11.0
Two and above	145	36.2
Place of ANC follow-up (*n* = 361)		
Health center	225	56.1
Hospital	99	24.7
Health post	14	3.5
Private clinic	23	5.7
Number of visits in ANC follow-up (*n* = 361)		
<4	170	42.4
≥4	191	47.6
Problems encountered during pregnancy		
Yes	53	13.2
No	348	86.8
Mode of delivery		
SVD	274	68.3
Instrumental delivery	43	10.7
Cesarean delivery	84	20.9
Qualification of labor attendant		
Doctor	129	32.2
Midwife/nurse	272	67.8

**Table 5:** Experience towards health care service and facilities, concerning their neonate at comprehensive and referral hospitals of southern Ethiopia, 2022 (*n* = 401).

Variables	Strongly disagree with *F* (%)	Disagree with *F* (%)	Uncertain with *F* (%)	Agree with *F* (%)	Strongly agree with *F* (%)
The child's room is quiet enough for him/her to rest	8 (2.0)	35 (8.7)	6 (1.5)	234 (58.7)	118 (29.4)
The caregivers take care of the infant to lay well-cared for bed	5 (1.2)	19 (4.7)	9 (2.2)	215 (54.6)	153 (38.2)
There is a provision of water and food by the hospital	8 (2.0)	54 (13.5)	15 (3.7)	219 (54.6)	105 (26.2)
There is the quality of food	17 (4.2)	81 (20.2)	61 (15.2)	181 (45.1)	61 (15.2)
There is a functional and clean toilet	17 (4.2)	106 (26.4)	28 (7.0)	200 (49.9)	50 (12.5)
There is ventilation in the room	20 (5.0)	96 (23.9)	23 (5.7)	176 (43.9)	86 (21.4)
There is adequate treatment	8 (2.0)	32 (8.0)	38 (9.5)	229 (57.1)	94 (23.4)
There is enough bed in this ward	13 (3.2)	62 (15.5)	60 (15.0)	163 (40.6)	103 (25.7)
The hospital compound is cleaned	5 (1.2)	45 (11.2)	45 (11.2)	241 (60)	65 (16.2)
There is an availability of necessary information using direction indicators	31 (7.7)	93 (23.7)	73 (18.2)	166 (41.4)	38 (9.5)
There is access to ways to present complaints to concerned bodies	7 (1.7)	65 (16.2)	37 (9.2)	233 (58.1)	59 (14.7)
Accessibility of necessary laboratory investigation	3 (0.7)	23 (5.7)	59 (14.7)	224 (55.9)	92 (22.9)
Availability of enough chairs in the waiting area	14 (3.5)	78 (19.5)	42 (10.5)	202 (50.4)	65 (16.2)

**Table 6:** Parental satisfaction towards care given at NICU in comprehensive and referral hospitals of southern Ethiopia, 2022 (*n* = 401).

Variables	Frequency	Percentage (%)
Receiving care	382	95.3
Listening to the parent's opinions about the child's needs	376	93.8
Opportunity to participate in discussions concerning the child's examination	320	79.8
Description of the child's expected outcome	376	93.8
Progress of baby's health condition	358	89.3
Safeness of NICU environment	393	98.0
Opportunity in asking questions of staff	386	96.3
Spending enough time at the child's bedside	370	92.3
Explanation of the treatment given	343	85.5
Encouragement and emotional support	353	88.0
Keeping well informed about the child's condition and procedure result	329	82.0
Respecting confidentiality	395	98.5
Consent and permission before procedures	287	71.6
Allowance to have visitors	292	72.8
Advising about ways to stay health	379	94.5
Counseling about breastfeeding techniques	385	96.0

**Table 7:** Factors associated with parental satisfaction towards care given at NICU incomprehensive and referral hospitals of southern Ethiopia, 2022 (*n* = 401).

Variables	Level of parental satisfaction		COR (95% CI)	AOR (95% CI)	*P* value
	Satisfied (%)	Unsatisfied (%)			
Educational status					
No formal	51 (44.0)	65 (56.0)	0.27 (0.15, 0.47)	0.15 (0.07, 0.31)	<0.001^∗^
Primary	60 (69.8)	26 (30.2)	0.78 (0.41, 1.47)	0.46 (0.21, 0.98)	0.044
Secondary	61 (66.3)	31 (33.7)	0.66 (0.36, 1.23)	0.57 (0.27, 1.17)	0.123
Collage and above	80 (74.8)	27 (25.2)	1	1	
Infant breastfeeding					
Yes	198 (66.4)	100 (33.6)	1.80 (1.13, 2.83)	1.20 (0.68, 2.11)	0.526
No	54 (52.4)	49 (47.6)	1	1	
Social support					
Strong	140 (74.1)	49 (25.9)	4.43 (2.51, 7.83)	1.82 (0.89, 3.72)	0.103
Moderate	83 (60.1)	55 (39.9)	2.34 (1.31, 4.17)	1.45 (0.72, 2.93)	0.304
Poor	29 (39.2)	45 (60.8)	1	1	
Mode of delivery					
SVD	168 (61.3)	106 (38.7)	1.03 (0.62, 1.70)	1.37 (0.74, 2.53)	0.314
Instrumental delivery	33 (76.7)	10 (23.3)	2.14 (0.93, 4.91)	3.49 (1.28, 9.51)	0.014
C.S	51 (60.7)	33 (39.3)	1	1	
Information about NICU rules					
Agree	213 (63.4)	123 (36.6)	1.15 (0.67, 1.99)	1.26 (0.64, 2.48)	0.502
Disagree	39 (60.0)	26 (40.0)	1	1	
Functionality and cleanness of toilet					
Agree	170 (68.0)	80 (32.0)	1.79 (1.18, 2.71)	1.52 (0.87, 2.65)	0.140
Disagree	82 (54.3)	69 (45.7)	1	1	
Information using direction indicator					
Agree	163 (79.9)	41 (20.1)	4.82 (3.10, 7.51)	3.14 (1.85, 5.31)	<0.001^∗^
Disagree	89 (45.2)	108 (54.8)	1	1	
Accessibility of ways to present complaints					
Agree	205 (70.2)	87 (29.8)	3.11 (1.97, 4.90)	1.43 (0.76, 2.70)	0.269
Disagree	47 (43.1)	62 (56.9)	1	1	
Availability of enough chairs in the waiting area					
Agree	200 (74.9)	67 (25.1)	4.71 (3.02, 7.34)	3.26 (1.81, 5.87)	<0.001^∗^
Disagree	52 (38.8)	82 (61.2)	1	1	

^∗^Statistically significant variable at *P* < 0.05.

## Data Availability

The data sets analyzed during the current study are available to all authors for reasonable request.

## Abbreviations

ANC: Antenatal care
BSC: Bachelor of science
FCC: Family-centered care
NICU: Neonatal intensive care unit
ODK: Open Data Kit
OSSS: Oslo social support scale
RMC: Respectful maternity care
SNNPR: Southern Nations, Nationalities, and People's Region
SPSS: Statistical Package for Social Science
WHO: World Health Organization.
